# Rectal prolapse traumatizes rectal neuromuscular microstructure explaining persistent rectal dysfunction

**DOI:** 10.1007/s00384-016-2649-8

**Published:** 2016-09-06

**Authors:** Matthias Kraemer, Werner Paulus, David Kara, Saskia Mankewitz, Stephanie Rozsnoki

**Affiliations:** 1 Department of General and Visceral Surgery, Coloproctology, St. Barbara-Klinik, Hamm, Germany; 2 Institute of Neuropathology, University-Hospital, Westfälische Wilhelms-Universität, Münster, Germany

**Keywords:** Rectal dysfunction, Megarectum, Rectal hyposensitivity, Rectal inertia, Obstructed defecation, Rectal prolapse

## Abstract

**Purpose:**

Internal rectal prolapse is common and correlates with age. It causes a plug-like physical obstruction and is a major cause of defecation disorder. The progressive distortion of the prolapsing rectum likely causes secondary defects in the rectal wall, which may exacerbate rectal dysfunction. We undertook a prospective observational study to detect and quantify the neurologic and histopathologic changes in the rectal wall.

**Methods:**

We examined dorsal and ventral rectal wall specimens from consecutive patients with internal rectal prolapse undergoing stapled transanal rectal resection (STARR). We subjected specimens to histopathologic and neuropathologic assessment, including immunohistochemistry. We also recorded patients’ clinical and demographic characteristics and sought correlations between these and the pathologic findings.

**Results:**

We examined 100 specimens. The severity of rectal prolapse and the extent of descent of the perineum correlated significantly with age. Concomitant hemorrhoidal prolapse was noted in all male patients and in 79 % of female patients. Muscular and neuronal defects were detected in 94 and 90 % of the specimens, respectively. Only four specimens (4 %) were free of significant structural defects.

**Conclusion:**

Rectal prolapse traumatizes the rectum causing neuromuscular defects. The tissue trauma is due to shearing forces and ischemia caused by the intussusception. This initiates a self-reinforcing vicious circle of physical and functional obstruction, further impairing rectal evacuation and causing constipation and incontinence. The correlation between extent of prolapse and age suggests that internal rectal prolapse can be considered a degenerative disorder. Neural and motor defects in the wall of the rectum caused by rectal prolapse are likely irreversible.

## Introduction

The last decade has brought a better understanding of acquired degenerative disorders of the rectum as well as new treatment options. In this context, internal rectal prolapse is recognized as a major cause of defecation disorder. The causal link between internal rectal prolapse and defecation disorder is largely accepted as a pathophysiologic concept known as obstructed defecation syndrome [1–12]. The cause of obstructed defecation is thought to be physical obstruction caused by the plug-like internal prolapse.

Imaging of patients with rectal prolapse—particularly dynamic defecography—often reveals an extremely flaccid and dilated rectum. Resection specimens of prolapsing rectum frequently appear fibrosed and scarred. It would be unlikely that a markedly flaccid, dilated, and intussuscepting rectum still retains the sensorimotor integrity required for evacuation. Conversely, a structurally normal rectum is unlikely to be flaccid enough to undergo intussusception.

The healthy rectum plays central sensory and evacuation roles in defecation. We hypothesized that the mechanics of internal rectal prolapse cause trauma to the rectal wall that in turn leads to sensorimotor impairment of rectal evacuation.

The confirmation of a causal relationship between prolapse, structural damage, and functional deficit would contribute to our understanding of sensorimotor disturbances of the rectum and might explain why dysfunction may persist despite operative removal of the obstructing prolapse.

The objective of this prospective observational study therefore was to detect and quantify histopathologic changes in rectal wall specimens taken from patients with rectal prolapse undergoing stapled transanal rectal resection (STARR). We sought changes that might indicate coexisting deficits of rectal sensory and motor functions and examined whether there was a correlation between structural changes, patients’ demographic characteristics, and clinical findings.

## Materials and methods

We undertook a prospective observational study, recording clinical data from patients with internal rectal prolapse undergoing STARR and subjecting resected specimens to detailed neuropathologic examination. We examined the extent to which defects were found in the mucosa-submucosa, muscularis propria, and connective tissue, as well as the neuronal plexus and cells of Cajal. We also sought correlations between defects, the sex, and age of the patients and clinical findings.

### Stapled transanal rectal resection

In western Europe, STARR has become a standard procedure for the resection of rectocele and to address internal rectal prolapse [1–12]. In carefully selected patients, the procedure is technically straightforward, safe, and generally has good functional outcomes. During surgery, the anterior and posterior rectal segments of the prolapse are usually resected separately and the remaining wound edges are stapled together. Generally, therefore, two separate full thickness specimens of the affected rectal wall are taken.

Although the STARR technique is widely accepted, it is still considered as controversial in some parts. However, possible pros and cons of the STARR procedure are not the issue of our study.

For the purposes of this study, we undertook investigations, made the diagnosis, selected patients for surgery, performed STARR, and managed patients postoperatively according to our current, routine clinical practice.

### Histologic and neuropathologic assessments

Formalin-fixed, paraffin-embedded samples of complete rectal wall were subject to histologic and neuropathologic assessments. All histologic assessment was undertaken at the Institute of Neuropathology of the University Hospital, Münster, Germany, according to previously published techniques [13, 14].

Specimens were stained with hematoxylin and eosin or Elastica van Gieson stains. Immunohistochemical staining was performed for the following antigens: CD117 (Clone c-Kit, Dako, Glostrup, Denmark, concentration 1:400, boiling pretreatment at pH 6.1), S100 (Dako, concentration 1:4000, no boiling pretreatment), calretinin (Dako, clone DAK-calret 1, concentration 1:50, boiling pretreatment at pH 9.0), and muscle actin (clone HHF35, Dako, concentration 1:400, boiling pretreatment at pH 6.1). All samples were independently assessed by two raters (WP and SR).

We examined for mucosa prolapse syndrome (manifested by fibromuscular obliteration, crypt atrophy, erosion, and ulceration) and assessed the integrity of the muscularis propria (for layering, atrophy of the circular or longitudinal layer, scarring of the circular or longitudinal layer, or inflammation), connective tissue (quantity and location), the submucosal plexus (for its structure), the myenteric plexus (for quantity of ganglion cells and scarring), the interstitial cells of Cajal (for density), and the general severity of pathology (see also supplemental Fig. 1).

**Fig. 1:**
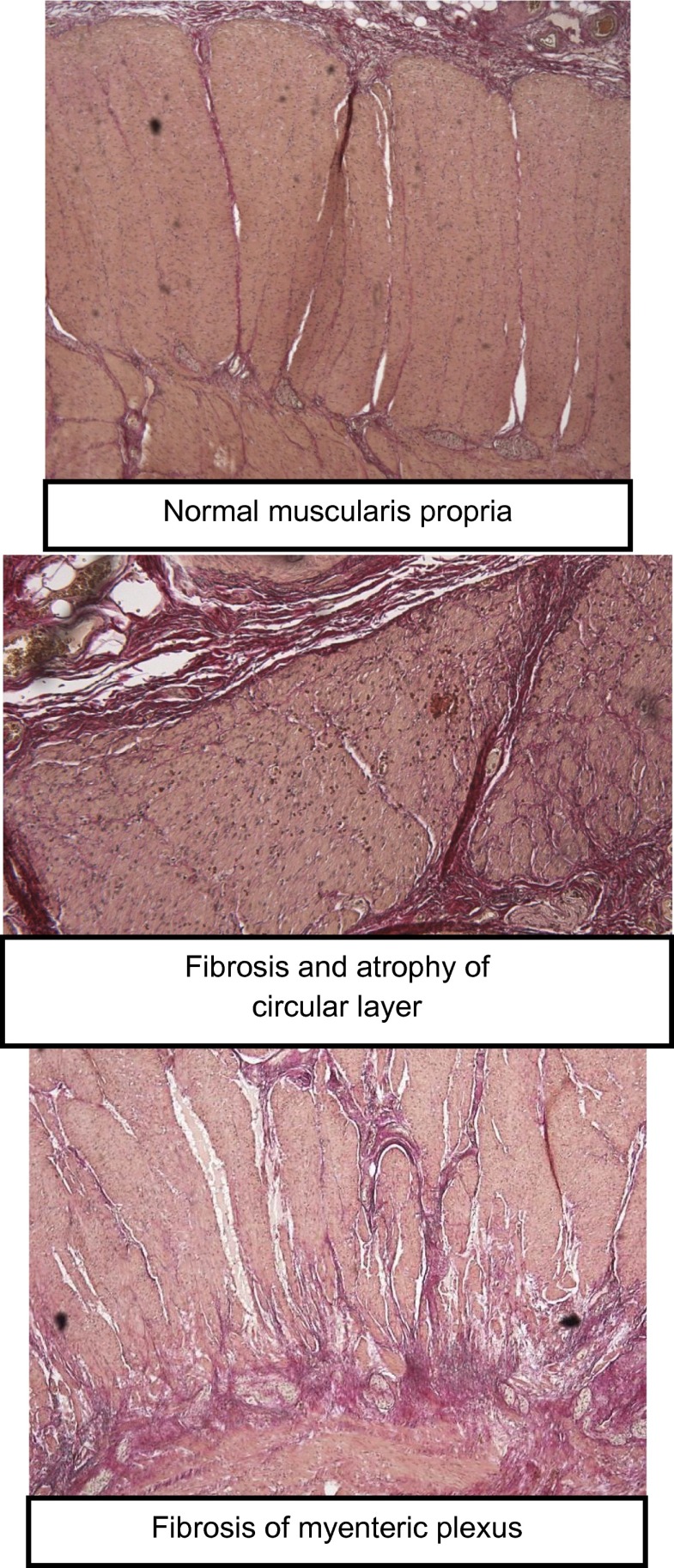
Examples of some of the histologic findings

### Statistical analysis

We used the Fisher’s exact test, the chi-squared test, and the Kruskal-Wallis test to compare the clinical and histopathologic features of the patients with rectal prolapse (SPSS software, version 20.0; IBM Corporation, Armonk, NY). *P* values <0.05 were considered statistically significant. Statistical support was provided by the Institute of Biostatistics and Clinical Research, Westfälische Wilhelms-Universität, Münster, Germany.

### Ethics approval

Conduct of the study was approved by the local ethics board (2013–629-f-S, Ethikkommission der Ärztekammer Westfalen-Lippe) and registered as a clinical study with our national database (DRKS00005662, Deutsches Register Klinische Studien).

## Results

We collected 100 specimens from 51 consecutive patients diagnosed with obstructed defecation and rectocele/rectal prolapsed, who underwent STARR following our routine preoperative therapeutic and diagnostic protocol (patient demographics; Table 1). Of these, 49 were ventral wall specimens and 51 dorsal wall specimens. Two patients in our cohort only required semicircular STARR, which therefore yielded only one specimen. Four patients who had a previous anorectal operation during the previous 12 months were excluded from the study. Staining was performed on all 100 specimens.

**Table 1:** Patients’ demographic characteristics

	Number	Age median/average/range (years)
Female	39	57/58/54–80
Male	12	51/52//39–72
Total	51	54/57/39–80

### Clinical characteristics

The majority of female patients had a history of vaginal delivery (64 %) and/or hysterectomy (44 %; Table 2). The severity of rectal prolapse and extent of the descending perineum corresponded significantly with age (Tables 3 and 4). Hemorrhoids were present in all male patients and in 74 % of female patients (Table 5). The vast majority of female patients were found to have rectocele (Table 6), again correlating with age.

**Table 2:** Obstetric and gynecologic histories of female patients

(*n* = 39)	Vaginal delivery	Hysterectomy
16 (41 %)	+	−
9 (23 %)	+	+
8 (21 %)	−	+
6 (15 %)	−	−

**Table 3:** Extent of rectal intussusception (RI) versus patient age

Rectal intussusception (RI)	Number	Median/average age (years)
RI reaches upper part of anal canal	6	49/49
RI reaches lower part of anal canal	22	54/53
RI reaches external anal ring	15	62/62
RI reaches beyond external anal ring	6	68/69
Not stated	2	
RI versus age		*p* = 0.01

**Table 4:** Extent of descending perineum versus patient age

Descending perineum (DP)	Number	Median/average age (years)
No significant DP	5	51/50
Descend above level of ischial tuberosities	14	50/49
Reaches level of ischial tuberosities	25	60/61
Descend beyond level of ischial tuberosities	5	66/69
Not stated	2	
DP versus age		*p* = 0.015

**Table 5:** Extent of hemorrhoid disease versus patient age

Associated hemorrhoidal disease (HD)	Number	Male	Female	Median/average age (years)
No significant HD	8	0	8	55/56
Grade I	9	2	7	60/60
Grade II	21	7	14	53/56
Grade III	8	2	6	59/60
Grade IV	3	1	2	60/61
Not stated	2	0	2	
HD versus age				n.s.

**Table 6:** Extent of anterior rectocele in female patients versus patient age

Anterior rectocele (AR; females)	Number	Median/average age (years)
No significant AR	3	49/52
Small (digital examination produces only slight bulging of rectovaginal wall (RVW) seen through the introitus)	1	56
Medium (digital examination produces prominent bulging of RVW seen through the introitus)	30	56/59
Advanced (digital examination produces bulging of RVW protruding externally through the introitus; RVW appears thin and fibrosed)	3	63/62
Not stated	2	
AR versus age		n.s.

### Histologic changes

Muscular and neuronal defects were detected in 94 and 90 % of the specimens, respectively (Table 6). Only four specimens from three patients were free of significant histologic or neuropathologic findings (Table 7). Notably, two of these three patients had unremarkable ventral specimens but were nevertheless found to have defects on the opposite side. Only 1 of the 51 patients was found to be free of significant muscular or neuronal defects in both STARR specimens.

**Table 7:** Summary of neuropathologic and histopathologic findings in specimens taken during stapled transanal resection of the rectum

Neuropathologic and histopathological defects	Number of specimens affected		
	Total	Ventral	Dorsal
Number of specimens	100	49	51
Crypt atrophy	14	8	6
Fibromuscular obliteration of lamina propria	14	10	4
Atrophy of circular layer	68	34	34
Fibrosis of circular layer	81	39	42
Atrophy of longitudinal muscle layer	35	14	21
Fibrosis of longitudinal muscle layer	65	29	36
Rarification of Cajal cells	27	14	13
Fibrosis of myenteric plexus	85	41	44
Neuropathological defects classified as “moderate” or “advanced”	49	25	24
No neuronal defects	10	6	4
No muscular defects	6	3	3
Neither neuronal nor muscular defects	4	3	1

## Discussion

Our findings support the hypothesis that internal rectal prolapse formation initiates a vicious circle of progressive structural damage and ensuing loss of function. Internal rectal prolapse is a plug-like intussusception of the rectum. The tissue trauma is most likely caused by shearing forces and ischemia caused by the intussusception.

The healthy rectum assumes an important sensory role in the physiology of defecation. There is consensus that increased intraluminal pressure in the rectum is perceived by receptors, although these have not been conclusively identified [15–19]. Above a certain threshold, rectal intraluminal pressure initiates the urge to defecate. The threshold for urge appears to be variable and may be influenced by acquired changes of the rectal wall (caused for example by inflammatory diseases, radiation, or acquired degenerative changes) [20–23].

Contrary to widely held beliefs, a physiologically intact rectum has no role as reservoir. However, with advancing age, acquired degenerative changes, and ensuing loss of evacuation strength, the rectum commonly mimics a stool-filled reservoir. The rectum acts as barrier to colonic peristalsis, which usually ends at the recto-sigmoid junction and is not propagated into the rectum [18]. During the resting phase, there appear to be retrograde propulsion motor complexes that transport smaller amounts of fecal matter back into the sigmoid colon until mass peristalsis from above is initiated [24]. Retrograde propulsive activity is also stimulated by the conscious suppression of defecation urge. A chronically stool-impacted rectum, which is a common finding, particularly in the elderly, can therefore be taken as evidence for a rectal evacuation deficit.

Evacuation of stool is also supported by active rectal motor activity. Defecation can be consciously initiated by the parasympathetic nervous system. To evacuate stool, the levator and anal sphincters relax, opening the anal canal. The rectum contracts mainly axially. To achieve axial contraction, colonic tenia spread anatomically over the rectum to form the external layer of the muscularis propria, which covers the entire organ. There is, however, also transverse peristalsis in the rectum brought about by contraction of the inner and more circular layer of the rectal muscularis propria [15–18].

**Fig. 2:**
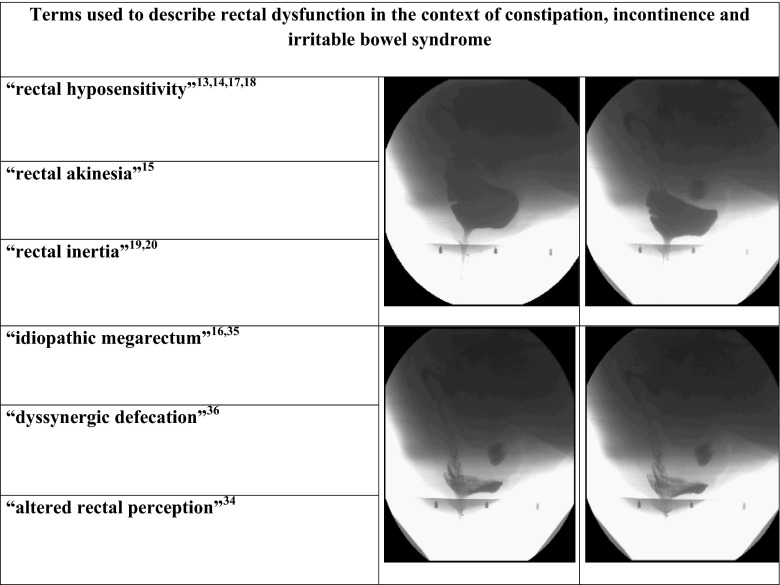
Defecographic sequence showing distortion of the rectal wall associated with internal rectal prolapse formation

The healthy rectum therefore has important and complex sensory and evacuation functions for defecation; proper sensory and motor functions require intact nerve and muscular structures in the wall of the rectum. Our findings confirm that rectal prolapse causes sufficient trauma to compromise these functions. This is perhaps not surprising if one considers the marked morphologic changes in an intussuscepting rectum. Dynamic defecography often reveals an extremely flaccid and dilated rectum in patients with rectal prolapse (Fig. 2).

Our finding that there is a positive correlation between age and the extent of internal rectal prolapse and rectocele strongly supports our opinion that the disorder is acquired and foremost a degenerative one. It may possibly be regarded as yet one more specific aspect of aging and be likened to other age-associated degenerative disorders.

It is likely that prolapse formation starts as mucosal prolapse and that in many cases, a reduction of connective tissue tautness may contribute to it. The shearing forces exerted by the passage of flatus or fecal matter push and pull the obstructing mucosal folds, thereby gradually involving and progressively traumatizing the deeper layers of the rectal wall and initiating a vicious circle of obstruction and prolapse formation.

Mucosal prolapse is in turn linked to many proctologic disorders, in particular hemorrhoidal disease. This is the rationale for undertaking stapled hemorrhoidopexy, as well as for ligation and injection sclerotherapy. Internal mucosal and ensuing rectal prolapse are therefore very common.

The formation of internal rectal prolapse invariably promotes physical and functional defecation obstruction. Obstruction may cause few symptoms, or even be asymptomatic, for many years. Obstruction usually evolves gradually, and the severity of its effects may ebb and flow, being influenced by factors such as diet, food allergies, laxative intake, and exercise. This explains why the obstructive effects of prolapse formation remain undetected in many patients and why the functional significance of these common degenerative changes is still a matter of much controversy.

That even near-to-complete obstruction of passage of fecal matter may cause few or no symptoms for a long time is not exclusive to patients with internal rectal prolapse. Patients with long-standing stenosing diverticular disease, Crohn’s disease, or malignancies often report “normal” bowel function.

The degenerative process is usually not limited to the infolding rectal wall; it appears to gradually involve the entire rectum and may affect the pelvic floor and adjoining organs, particularly in women. This typically presents with associated phenomena such as a descending perineum. As the diameter of the pelvic outlet is smaller in men, a smaller amount of mucosal prolapse—as seen in hemorrhoidal disease—may result in significant obstruction.

There may be factors contributing to the development of internal rectal prolapse or rectocele, such as defecation disorders or a history of vaginal delivery and/or hysterectomy in women; however, numerous patients are affected without any obvious risk factors.

The acquired structural damage leads to impairment of rectal function, which may persist despite operative repair of the obstructing prolapse. The persistence of rectal dysfunction after surgery contributes to the ongoing debate about the optimal intervention for rectal prolapse. For many decades, this has been a matter of lively debate. It appears likely that the mode of operative repair, in particular whether the rectum is mobilized anteriorly, posteriorly, or circumferentially, may have little or no effect on functional outcome. Within the context of circular mobilization, the significance of the so-called lateral ligaments of the rectum has in particular been a matter of some controversy [25]. Whatever nerves these ligaments may contain, however, they may be of negligible significance in a prolapsing and therefore dysfunctional rectum.

The self-reinforcing sequence of physical and functional obstruction of the rectum may also explain why there have been consistent reports of marked motor and sensory disturbances of the rectum detected in patients with constipation, defecation obstruction, fecal incontinence, and irritable bowel syndrome (Fig. 2) [26–36].

Internal rectal prolapse is common, and therefore, rectal dysfunction is common. It is an often unrecognized and possibly to a certain extent unavoidable consequence of aging that affects men and women alike. It initiates a vicious circle of physical obstruction and functional impairment. Progressive impairment of rectal evacuation and the ensuing fecal congestion cause the typical symptom overlap of constipation and incontinence. The current strategy of evaluating and treating constipation and fecal incontinence as two distinct disorders needs to be reviewed in the light of this.

An understanding that rectal evacuation depends on an intact rectum, and that over time, the rectum is as prone as any other organ to age-related progressive degeneration in structure and function, increases our understanding of constipation and incontinence—particularly in the elderly. Both disorders significantly impair quality of life and are also of considerable socioeconomic relevance.

## References

[1] Arroyo A, Pérez-Vicente F, Serrano P, Sánchez A, Miranda E, Navarro JM, Candela F, Calpena R (2007). Evaluation of the stapled transanal rectal resection technique with two staplers in the treatment of obstructive defecation syndrome. J Am Coll Surg.

[2] Arroyo A, González-Argenté FX, García-Domingo M, Espin-Basany E, De-la-Portilla F, Pérez-Vicente F, Calpena R (2008). Prospective multicentre clinical trial of stapled transanal rectal resection for obstructive defaecation syndrome. Br J Surg.

[3] Boccasanta P, Venturi M, Salamina G, Cesana BM, Bernasconi F, Roviaro G (2004). Int J Color Dis.

[4] Corman ML, Carriero A, Hager T, Herold A, Jayne DG, Lehur PA, Lomanto D, Longo A, Mellgren AF, Nicholls J, Nyström PO, Senagore AJ, Stuto A, Wexner SD (2006). Consensus conference on the stapled transanal rectal resection (STARR) for disordered defaecation. Color Dis.

[5] Ellis CN (2007). Stapled transanal rectal resection (STARR) for rectocele. J Gastrointest Surg.

[6] Frascio M, Stabilini C, Ricci B, Marino P, Fornaro R, De Salvo L, Mandolfino F, Lazzara F, Gianetta E (2008). Stapled transanal rectal resection for outlet obstruction syndrome: results and follow-up. World J Surg.

[7] Ganeshan A, Anderson EM, Upponi S, Planner AC, Slater A, Moore N, D’Costa H, Bungay H (2008). Imaging of obstructed defecation. Clin Radiol.

[8] Ommer A, Albrecht K, Wenger F, Walz MK (2006). Stapled transanal rectal resection (STARR): a new option in the treatment of obstructive defecation syndrome. Langenbeck’s Arch Surg.

[9] Pechlivanides G, Tsiaoussis J, Athanasakis E, Zervakis N, Gouvas N, Zacharioudakis G, Xynos E (2007). Stapled transanal rectal resection (STARR) to reverse the anatomic disorders of pelvic floor dyssynergia. World J Surg.

[10] Schwandner O, Fürst A (2008). Aktueller Stellenwert der transanalen Stapler-Resektion des distalen Rektums (STARR) bei Obstruktivem Defäkations-Syndrom. Zentralbl Chir.

[11] Schwandner O, Stuto A, Jayne D, Lenisa L, Pigot F, Tuech JJ, Scherer R, Nugent K, Corbisier F, Basany EE, Hetzer FH (2008). Decision-making algorithm for the STARR procedure in obstructed defecation syndrome: position statement of the group of STARR pioneers. Surg Innov.

[12] Slim K, Mezoughi S, Launay-Savary MV, Tuech JJ, Michot F, Sielezneff I, Sastre B, Pigot F, Juguet F, Faucheron JL, Voirin D, Chipponi J (2008). Traitement de la rectocèle par résection rectale transanale à la pince mécanique: résultats à moyen terme d’une étude multicentrique en France. J Chir.

[13] Knowles CH, De Giorgio R, Kapur RP, Bruder E, Farrugia G, Geboes K, Gershon MD, Hutson J, Lindberg G, Martin JE, Meier-Ruge WA, Milla PJ, Smith VV, Vandervinden JM, Veress B, Wedel T (2009). Gastrointestinal neuromuscular pathology: guidelines for histological techniques and reporting on behalf of the Gastro 2009 International Working Group. Acta Neuropathol.

[14] Stolte M, Rüschoff J, Klöppel G 2013 (Publ). Gastrointestinal neuromuscular pathology in chronic constipation; diverticular disease is associated with an enteric neuropathy as revealed by morphometric analysis; desmosis of the colon: a working hypothesis of primary chronic constipation Verdauungstrakt und Peritoneum (Book). Springer Verlag ISBN 978–3–642-02321-7. doi: 10.1007/978–3–642-02322-4

[15] Henry MM (1985). (Hg). Coloproctology and the pelvic floor.

[16] Mazier WP (1995). (Hg). Surgery of the colon, rectum, and anus.

[17] Nicholls RJ, Dozois RR (1997). (Hg). Surgery of the colon & rectum.

[18] Pulit S, Lunniss PJ, Scott SM (2012). The physiology of human defecation. Dig Dis Sci.

[19] Sabate JM, Coffin B, Jian R, Le Bars D, Bouhassira D (2000). Rectal sensitivity assessed by a reflexologic technique: further evidence for two types of mechanoreceptors. Am J Physiol Gastrointest Liver Physiol.

[20] van Nieuwenhoven MA, Kilkens TO (2012). The effect of acute serotonergic modulation on rectal motor function in diarrhea-predominant irritable bowel syndrome and healthy controls. Eur J Gastroenterol Hepatol.

[21] Krol R, Hopman WP, Smeenk RJ, Van Lin EN (2012). Increased rectal wall stiffness after prostate radiotherapy: relation with fecal urgency. Neurogastroenterol Motil.

[22] Sloots CE, Felt-Bersma RJ, West RL, Kuipers EJ (2005). Stimulation of defecation: effects of coffee use and nicotine on rectal tone and visceral sensitivity. Scand J Gastroenterol.

[23] Yeoh EK, Bartholomeusz DL, Holloway RH, Fraser RJ, Botten R, Di Matteo A, Moore JW, Schoeman MN (2010). Disturbed colonic motility contributes to anorectal symptoms and dysfunction after radiotherapy for carcinoma of the prostate. Int J Radiat Oncol Biol Phys.

[24] Rao S, Welcher K (1996). Periodic rectal motor activity: the intrinsic colonic gatekeeper?. Am J Gastroenterol.

[25] Charran O, Muhlemann M, Shah S, Tubbs RS, Loukas M (2014). Ligaments of the rectum: anatomical and surgical considerations. Am Surg.

[26] Burgell RE, Lelic D, Carrington EV, Lunniss PJ, Olesen SS, Surguy S, Drewes AM, Scott SM (2013). Assessment of rectal afferent neuronal function and brain activity in patients with constipation and rectal hyposensitivity. Neurogastroenterol Motil.

[27] Burgell RE, Bhan C, Lunniss PJ, Scott SM (2012). Fecal incontinence in men: coexistent constipation and impact of rectal hyposensitivity. Dis Colon rectum.

[28] Faucheron JL, Dubreuil A (2000). Rectal akinesia as a new cause of impaired defecation. Dis Colon rectum.

[29] Gladman MA, Williams NS, Scott SM, Ogunbiyi OA, Lunniss PJ (2005). Medium-term results of vertical reduction rectoplasty and sigmoid colectomy for idiopathic megarectum. Br J Surg.

[30] Knowles CH, Thin N, Gill K, Bhan C, Grimmer K, Lunniss PJ, Williams NS, Scott SM (2012). Prospective randomized double-blind study of temporary sacral nerve stimulation in patients with rectal evacuatory dysfunction and rectal hyposensitivity. Ann Surg.

[31] Lee TH, Lee JS, Hong SJ, Jeon SR, Kwon SH, Kim WJ, Kim HG, Cho WY, Cho JY, Kim JO, Lee JS (2013). Rectal hyposensitivity and functional anorectal outlet obstruction are common entities in patients with functional constipation but are not significantly associated. Korean J Intern Med.

[32] Scott SM, van den Berg MM, Benninga MA (2011). Rectal sensorimotor dysfunction in constipation. Best Pract Res Clin Gastroenterol.

[33] Shafik A, Ahmed I (2002). Study of the motile activity of the colon in rectal inertia constipation. J Gastroenterol Hepatol.

[34] Posserud I, Syrous A, Lindström L, Tack J, Abrahamsson H, Simrén M (2007). Altered perception in irritable bowel syndrome is associated with symptom severity. Gastroenterology.

[35] Williams NS, Fajobi OA, Lunniss PJ, Scott SM, Eccersley AJ, Ogunbiyi OA (2000). Vertical reduction rectoplasty: a new treatment for idiopathic megarectum. Br J Surg.

[36] Woodward S, Norton C, Chiarelli P (2014). Biofeedback for treatment of chronic idiopathic constipation in adults. Cochrane Database Syst Rev.

